# Circulating tumor cell profiling for precision oncology

**DOI:** 10.1002/1878-0261.12901

**Published:** 2021-02-01

**Authors:** Mahmoud Labib, Shana O. Kelley

**Affiliations:** ^1^ Department of Pharmaceutical Sciences University of Toronto Canada; ^2^ Institute for Biomaterials and Biomedical Engineering University of Toronto Canada; ^3^ Department of Biochemistry University of Toronto Canada; ^4^ Department of Chemistry University of Toronto Canada

**Keywords:** CTC enrichment, drug selection, microfluidics, molecular profiling, precision oncology, tumor resistance

## Abstract

Analysis of circulating tumor cells (CTCs) collected from patient's blood offers a broad range of opportunities in the field of precision oncology. With new advances in profiling technology, it is now possible to demonstrate an association between the molecular profiles of CTCs and tumor response to therapy. In this Review, we discuss mechanisms of tumor resistance to therapy and their link to phenotypic and genotypic properties of CTCs. We summarize key technologies used to isolate and analyze CTCs and discuss recent clinical studies that examined CTCs for genomic and proteomic predictors of responsiveness to therapy. We also point out current limitations that still hamper the implementation of CTCs into clinical practice. We finally reflect on how these shortcomings can be addressed with the likely contribution of multiparametric approaches and advanced data analytics.

## Graphical abstract

This Review highlights the emerging role of circulating tumor cell (CTC) profiling in precision oncology. We summarize key technologies currently employed for CTC isolation and analysis and discuss recent clinical studies that examined CTCs for genomic and proteomic predictors of therapeutic response. We point out challenges associated with implementation of CTC profiling into clinical practice and reflect on potential solutions.

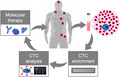

**Abbreviations**
ADT: androgen deprivation therapy
cfDNA: circulating cell‐free DNA
cfRNA: circulating cell‐free RNA
CGH: comparative genomic hybridization
CNG, copy number gain ; CNVs: copy number variations
CTC: circulating tumor cell
CTSC: circulating tumor stem cell
DEF: dielectrophoresis
DFF: dean flow fractionation
EMT: epithelial‐to‐mesenchymal transition
EVs: extracellular vesicles
FACS: fluorescence‐activated cell sorting
FISH: fluorescence *in situ* hybridization
GEDI: geometrically enhanced differential immunocapture
ITH: intra‐tumoral heterogeneity
LDT: laboratory‐developed test
MACS: magnetic‐activated cell sorting
MagRC: magnetic ranking cytometry
mCRPC: metastatic castration‐resistant prostate cancer
MET: mesenchymal‐to‐epithelial transition
MICs: metastasis‐initiating cells
MRD: minimal residual disease
NSCLC: non‐small‐cell lung cancer
PDAC: pancreatic ductal adenocarcinoma
PET‐CT: positron emission tomography‐computed tomography
qRT‐PCR: quantitative real‐time polymerase chain reaction
SARMS: scorpion amplification refractory mutation system
SCBC: single‐cell barcode chip
SCLC: small cell lung cancer
scWestern: single‐cell Western blotting
SERD: selective estrogen receptor downregulator
TCGA: The Cancer Genome Atlas
TEPs: tumor‐educated platelets
TKI: tyrosine kinase inhibitor

## 1 Introduction

One of the key objectives of precision oncology is to improve diagnosis and treatment of cancer [1]. The Cancer Genome Atlas (TCGA) research network has provided comprehensive molecular profiles for hundreds of tumors, which allowed the recognition of different molecular aberrations and their functional roles in different tumors as well as the identification of potential druggable targets, which is vital for the realization of precision oncology [2]. As a result, we have witnessed an explosion of cancer therapeutics that target specific molecular aberrations driving tumors, interfere with signaling pathways, or block immune checkpoints [3].

Precision oncology has been slowly but surely changing the traditional concept of ‘one treatment fits all’ to ‘one patient, one treatment’, which heightened the need for robust molecular profiling methods for individual patients' tumors, often obtainable only through image‐guided tissues biopsies [4, 5]. However, the complex nature of tumor biology usually brings about a substantial degree of heterogeneity, which requires comprehensive analysis of the primary tumor as well as repeated sampling of its metastatic lesions. This is not feasible in clinical practice because tissue biopsies are often restricted to few sampling points and accessible sites due to the associated cost, risks and logistical hurdles [6]. Also, tumor tissue sampling might seed the tumor cells around the sampling area, leading to local dissemination [7]. The risks and caveats of solid biopsies become more pronounced when dealing with multiple metastases that require sampling from different metastatic lesions and comparing their molecular profiles to obtain a holistic view of the disease. Unfortunately, a growing body of evidence suggests that the molecular profiles of primary tumors and their metastatic lesions are not always concordant, owing to intrinsic tumor heterogeneity [8]. These pitfalls have gradually turned the tide of precision oncology toward liquid biopsies because the blood contacts most tumor sites, and thus, it became logical to speculate that liquid biopsies would better mirror heterogeneous tumors than tissues biopsies [9].

In contrast to tissue sampling, liquid biopsies are not limited by sampling frequency, tumor accessibility or the presence of clinically overt disease. The term liquid biopsy refers to sampling and detecting analytes in different biological fluids, such as blood, urine, saliva, cerebrospinal fluid, ascites, and pleural effusions [10, 11]. Analytes in the peripheral blood of cancer patients typically contain circulating tumor cells (CTCs), circulating cell‐free DNA (cfDNA), circulating cell‐free RNA (cfRNA), extracellular vesicles (EVs), tumor‐educated platelets (TEPs), proteins, and other metabolites [12]. Among these analytes, CTCs represent the most prominent liquid biopsy marker that have been broadly utilized to provide information about features of primary tumors or metastases, track evolutionary dynamics and heterogeneity of tumors, and detect the emergence of treatment resistance, residual disease, and recurrence [13]. Profiling CTCs can provide a ‘moving picture’ of longitudinal tumor progression, whereas only a ‘snapshot’ of the cancer can be gained by a conventional tissue biopsy [14].

Response to cancer therapy is conventionally determined based on diagnostic imaging and blood‐based tumor biomarkers. Diagnostic imaging techniques such as positron emission tomography‐computed tomography (PET‐CT) suffer from low sensitivity and specificity [15]. Analysis of tumor biomarkers is also hampered by low sensitivity and specificity. In addition, sampling patients with low tumor burden might lead to false negative results, whereas false‐positive findings might arise from the release of biomarkers by noncancerous tissues and comorbid diseases [16]. One of the key advantages of using CTCs as a molecular proxy to monitor cancer resistance is that they can provide quantitative information on treatment response and allow qualitative elucidation of resistance mechanisms. Unfortunately, capturing CTCs from blood samples has been technically challenging due to their low abundance (a few to hundreds per milliliter) in a high background (billions per milliliter) of blood cells [17]. With the recent advances in single‐cell technologies made during the last 5 years, profiling CTCs at the single‐cell level in peripheral blood has offered a unique minimally invasive approach to track therapeutic resistance [18].

In this Review, we survey recent studies that have leveraged CTC‐level information to predict tumor response to therapy. We discuss current limitations that still hamper the implementation of CTCs into clinical practice. We conclude by reflecting on how these shortcomings might be addressed, and in particular, on the likely contributions of multiparameter assays and advanced data analytics.

## 2 Tumor resistance as a challenge in precision oncology

In precision medicine, the decision to treat with targeted therapy is dictated by the presence of a specific biomarker (e.g., gene mutation or amplification) in the tumor. Unfortunately, the response of many solid tumors to targeted therapy is < 50%, owing to tumor resistance, tumor heterogeneity, or inaccuracies produced by the choice of profiling method [19]. Resistance to targeted therapy can be classified as primary or secondary resistance. Primary or intrinsic resistance describes cancer patients who do not respond to treatment at all, whereas secondary or acquired resistance involves patients who initially respond to treatment then later fail to respond as the drug‐sensitive tumor cells die and the resistant subclones continue to grow. A case in point is BRAF^V600E^ melanoma patients who initially respond to treatment with tyrosine kinase inhibitors (e.g., vemurafenib); then, most of these patients relapse with a deadly disease due to secondary resistance [20].

Intratumor heterogeneity plays a major role in resistance to targeted therapy because a highly heterogeneous tumor would more likely harbor drug‐resistant subclones [21]. Although most targeted therapies are directed toward specific genetic aberrations, there are other factors that can contribute to resistance including epigenetic alterations that arise rapidly due to microenvironmental factors [22]. The surrounding tumor microenvironment can also contribute to tumor resistance by protecting the tumor cells from infiltrating drugs either by providing a physical barrier or through releasing paracrine signaling factors that can alter tumor cell survival [23]. This can lead to secondary resistance as the surviving tumor cells continue to grow under altered selective pressure [19]. Even exposure to therapy can impose selective pressure on the tumor, leading to the expansion of pre‐existing drug‐tolerant subclones that have survived initial therapy due to adoptive activation of alternative molecular pathways [24, 25]. Tumor resistance to therapy, whether it is intrinsic or acquired, is best studied using liquid biopsy markers to monitor tumor evolution under the selective pressure imposed by cancer therapy.

## 3 CTC biology and clinical implications

During metastasis, epithelial tumor cells undergo epithelial‐to‐mesenchymal transition (EMT), lose their polarity and secrete specific enzymes (e.g., cathepsin D) to digest the extracellular matrix and acquire migratory properties. Invasive tumor cells then reach the bloodstream and become CTCs. Subsequent to extravasation, CTCs can invade a new organ and undergo mesenchymal‐to‐epithelial transition (MET) [26]. There is substantial evidence that metastases can be driven by a population of CTCs with stem cell‐like features, such as self‐renewal and multipotentiality, hence referred to as circulating tumor stem cells (CTSCs) [27].

### 3.1 CTCs and resistance to therapy

The presence of CTCs with an EMT phenotype has been correlated with tumor relapse and resistance to therapy. For example, overexpression of CSV and plastin 3 in EMT‐positive CTCs was found to cause drug resistance in patients with metastatic colorectal cancer [28]. The acquisition of stem cell features during EMT was found to enhance the survival of the CTCs, leading to higher migratory and invasive properties as well as resistance to cancer therapy [29]. Also, the presence of CTCs with stem cell features can lead to tumor relapse following targeted therapy, such as in colorectal cancer [30]. This is because drug‐resistant CSCs might act as metastasis‐initiating cells (MICs), leading to more aggressive tumors due to the selection pressure imposed by the anticancer drug. Phenotypic switching of CTCs to a less differentiated phenotype has also been reported to correlate with tumor relapse after targeted therapy in melanoma patients [31], and the role of phenotypic switching in tumor resistance to therapy has also been reported in other types of cancer, such as breast, prostate, pancreatic, and non‐small‐cell lung cancer (NSCLC) [32]. The major mechanisms of therapeutic resistance in primary tumor cells have also been observed in CTCs, including target inactivation or mutation, improving genomic DNA repair, accelerated drug efflux, upregulation of quiescence markers, downregulation of proliferative markers, and suppression of oxygen radical formation [33]. The hypothesis that CTCs with EMT and/or stem cell phenotype represent the only route for initiating metastasis has been challenged by the finding that tumor cells can detach from the primary tumor and circulate in the blood as clusters of small cells (i.e., groups of > 2 CTCs and up to large micro‐emboli) [34]. The presence of CTCs clusters might also contribute to the metastatic potential and resistance to therapy. The ability of CTC clusters to evade cytotoxic drugs has been ascribed to lack of proliferation as indicated by Ki67 staining [35]. For instance, the overall survival after systemic therapy was significantly reduced in pancreatic ductal adenocarcinoma (PDAC) patients harboring large number of CTCs clusters (more than 30 clusters per 2 mL of blood) [36].

**Fig. 1:**
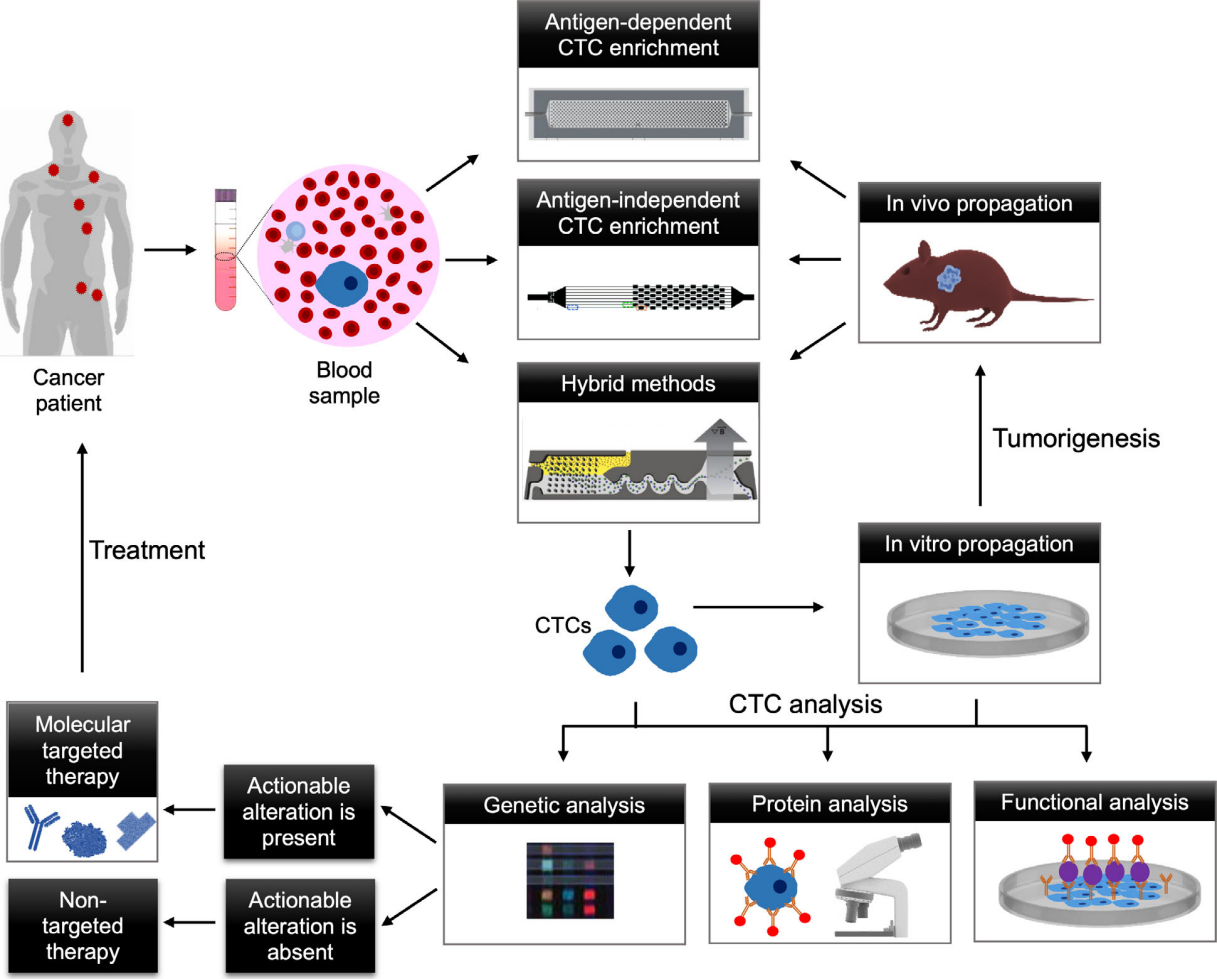
CTC analysis guiding precision oncology. To achieve this goal, CTCs are isolated from the blood of a cancer patient using antigen‐dependent or antigen‐independent CTC enrichment approaches or hybrid methods. The isolated CTCs can either be analyzed directly with single‐cell approaches or cultured *in vitro* before analysis. The CTCs propagated *in vitro* can also be xenografted to propagate *in vivo* then isolated using the previous approaches. The CTCs are analyzed for predictors of responsiveness to therapy using genetic, protein, and functional assays. Upon detection of actionable alterations, the patient is treated with molecular targeted therapy. In absence of molecular alterations, an appropriate therapeutic intervention is decided by the physician.

Expansion of CTCs from patients as cell lines or cell line‐derived xenografts might provide further clues about their therapeutic resistance and offer an opportunity to test drugs for personalized medicine. Unfortunately, CTC‐derived cell lines or xenografts require high CTC counts and might take several months, which make them unsuited for clinical practice [37]. In addition, long‐term passage of CTCs on a plate is likely to cause irreversible adaptation and clonal outgrowth [9]. Alternatively, isolation of CTCs and their downstream analysis, particularly at the single‐cell level, for molecular predictors of responsiveness to therapy can address these limitations and guide personalized oncology (Fig. 1). In the following section, we discuss CTC enrichment and analysis technologies, focusing on the approaches that have been successfully used for monitoring tumor resistance and informing drug selection.

### 3.2 CTC enrichment strategies

Methods used to enrich CTCs are divided into antigen‐dependent, antigen‐independent approaches or a combination of both. These methods have been comprehensively reviewed elsewhere [38, 39] but a brief summary of the methods that relate to the focus of this article is provided below.

#### 3.2.1 Antigen‐dependent CTC enrichment

This approach is usually performed using immunoaffinity techniques that utilizes a capture agent for positive selection of CTCs by targeting cell‐surface markers [40, 41], or negative selection via depletion of white blood cells (WBCs) [42].

##### Positive enrichment

Based on positive enrichment, the CellSearch platform is the only one to date that is approved by the Food and Drug Administration (FDA) for CTC enumeration [43]. In this method, the patient's blood is collected in a CellSave tube, which contains an anticoagulant and a proprietary fixative/stabilizer to preserve the blood for up to 96 h. The RBCs are removed by Ficoll centrifugation, and the buffy coat is then incubated with an EpCAM antibody conjugated with magnetic nanoparticles. Magnetically labeled CTCs are then isolated by applying a magnetic field and immunostained using a three‐color protocol for cytokeratin (CK) proteins, leukocyte antigen (CD45), and the nuclear stain (DAPI). The cells suspended in the magnetic chamber (MAGNEST) are then imaged with a fluorescence microscope. CTCs are identified by a distinct staining pattern (CK^+^/CD45^−^/DAPI^+^) and enumerated [44]. Magnetic‐activated cell sorter (MACS) is another commercially available platform for CTC isolation that relies on positive selection of CTCs [45]. However, both approaches suffer from low CTC recovery.

**Fig. 2:**
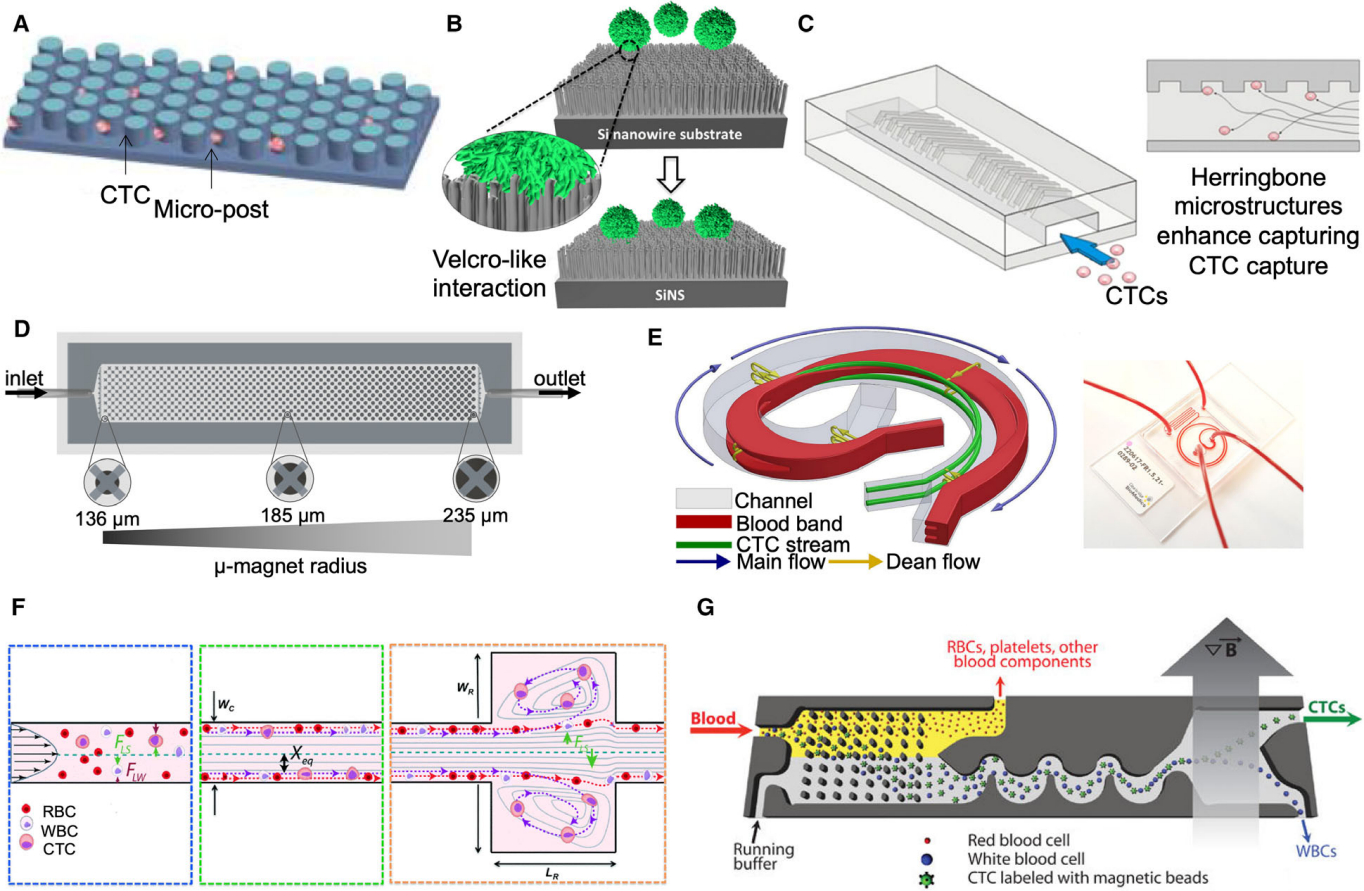
CTC enrichment approaches used for monitoring tumor resistance. (A–D) Antigen‐dependent CTC enrichment approaches. (A) Illustrative cartoon showing CTCs captured against the microposts within the CTC‐Chip. (B) The working mechanism of the nanoVelcro chip, which contains silicon nanowire substrate (SiNS) coated with EpCAM antibodies to enhance the capture efficiency of CTCs. (C) Illustration of the herringbone device (^HB^CTC‐Chip), featuring a microfluidic array of channels with a single inlet and outlet. (D) The microfluidic device used for magnetic ranking cytometry (MagRC) consists of 100 distinct capture zones. An array of X‐shaped microstructures creates regions of low flow velocity and circular nickel micromagnets patterned within the channel enhance the externally applied magnetic field. (E, F) Antigen‐independent CTC enrichment methods used for resistance monitoring. (E) Schematic illustration of Dean flow fractionation (DFF) principle of the antigen‐independent ClearCell Fx system (left). The target cells travel to the inner together with the blood band following the Dean flow stream in the first half curvature of the channel. Once the inner side of the channel is reached, the target cells remain focused while the blood band travels to the outer side in the second half curvature of the channel. Both target cells and nontarget cells are collected from different outlets at the end of the spiral channel. The microfluidic device utilized in the ClearCell Fx system (right) has sample and sheath inlets at one end and recovery and waste outlets at the other end. (F) Schematic of the Vortex chip featuring 8 channels. (a) At the channel inlet, the cells are randomly distributed and experience to opposing lift forces, the wall effect (*F*_LW_) and the shear‐gradient lift force (*F*_LS_). (b) As a result, the cells migrate to dynamic lateral equilibrium position (*X*_eq_), based to the channel cross section. (c) Upon entering the reservoir, the wall effect is reduced so large cells which still experience large *F*_LW_ are pushed to the vortices where they become captured, whereas small cells, which do not experience sufficient *F*_LW_ remain in the main flow. (G) Schematic of a hybrid CTC enrichment system, the CTC‐iChip. The blood is mixed with immunomagnetic beads and buffer before introduced into the device. CTCs are captured subsequent to inertial focusing and magnetophoresis. Figure A is reproduced with permission from Ref.[46]; Figure B is reproduced with permission from Ref. [47]; Figure C is reproduced with permission from Ref. [48]; Figure D is reproduced with permission from Ref. [41]. Figures E is reproduced with permission from Ref. [71]; Figure F is reproduced with permission from Ref. [72]; Figure G is reproduced with permission from Ref. [77]. Figure reproduced from Refs [41, 46, 47, 48, 71, 72, 77].

Several affinity‐based microfluidic devices have been developed for positive enrichment of CTCs. Microfluidics‐enabled immunoseparation involves capturing CTCs within antibody‐coated microfluidic devices. For example, the CTC‐Chip contains 78 000 microposts that are chemically conjugated with EpCAM antibody to isolate CTCs from blood (Fig. 2A) [40, 46]. The NanoVelcro chip contains a silicon nanowire substrate (SiNS) conjugated with EpCAM antibody via biotin‐streptavidin coupling (Fig. 2B) [47]. The nanostructured SiNS enhances the cell capture efficiency. The herringbone device, ^HB^CTC‐Chip, comprises herringbone microstructures to enhance the interaction between CTCs and the antibody‐coated surface via convective mixing (Fig. 2C) [48].

The captured CTCs can be released from the device by trypsinization, which might cause loss of cell‐surface receptors and subsequently confound further downstream analysis [49]. This downside has been addressed by designing smaller microfluidic devices with magnetic pores in which the CTCs are released by vertical flow centrifugation [50]. Newer iterations of the nanoVelcro device exploit competitive binding or a thermo‐responsive polymer substrate to facilitate the release of the captured CTCs. Also, nucleic acid aptamers have been used in some microfluidic sorting techniques instead of antibodies, due to their low cost, ease of modification, and simple release mechanism [51, 52].

Immunomagnetic separation involves prelabeling the cells with magnetic beads‐conjugated antibodies, followed by capturing CTCs within a microfluidic device exposed to a magnetic field [41]. This workflow has been adopted in the magnetic ranking cytometry (MagRC) technology, which can rank the cells according to the expression of cell‐surface proteins [53], intracellular proteins [54], or mRNAs [55]. The MagRC devices contain capture zones to rank the cells according to their magnetic loading (Fig. 2D). Cells with high magnetic loading are captured in earlier zones, whereas cells with low magnetic loading are captured in later zones. The cells can be simply retrieved by removing the magnetic field. Several microfluidic platforms have also exploited magnetically labeled CTCs to improve their detection speed and sensitivity, such as MagSweeper [56], IsoFlux [57], VerIFAST [58], and magnetic sifters [59].

The efficiency of CTC affinity selection is influenced by the selectivity of the antibody used for enrichment. Some antibodies are restricted to specific cancer types (e.g., EGFR‐, HER2‐expressing tumors) or could suffer from low selectivity because their target cell‐surface markers are also expressed by other cells (e.g., VCAM‐1, ICAM‐1) [60]. This limitation has been addressed by using antibody cocktails specific to several cell‐surface markers to capture all CTC subsets [48]. For example, the Adnatest is based on a combination of magnetic‐labeled antibodies specific to EpCAM, EGFR, and MUC1 [61]. The cytapheresis technology (e.g., Cell‐Collector) was introduced to overcome the problems of low CTC count in blood samples and of CTC stability during storage [62]. Cytapheresis is an invasive approach in which the blood is passed through a machine that retains EpCAM‐positive CTCs and returns the CTC‐depleted blood to the patient's bloodstream [63]. However, this method is time‐consuming and cannot be used repeatedly due to the possible contamination of blood with excess iron leaked from the magnetic nanoparticles/wires used for sorting [64].

##### Negative enrichment

Negative CTC selection has been sought to overcome the limitations of positive enrichment, such as heterogeneous expression of cell‐surface markers due to EMT and expression of the hallmark marker, EpCAM, by noncancerous cells in some diseases (e.g., benign colon disease). In this approach, magnetic beads conjugated with antibodies specific to CD45 and CD61 are used to deplete leukocytes and platelets, respectively [65]. However, negative selection is often followed by the isolation of circulating CD45^−^ endothelial cells to improve the recovery of CTCs.

Overall, antigen‐dependent CTC enrichment has several advantages but might not be the method of choice if the binding between the antibody and cell‐surface receptor leads to activation of specific signaling pathways, which might complicate further analysis of intracellular proteins or mRNAs [66]. In such case, a label‐free approach that relies on the physical properties of CTCs would be a better choice.

#### 3.2.2 Antigen‐independent CTC enrichment

Antigen‐independent, also called ‘label‐free’, CTC isolation approaches are used to isolate CTCs from the blood, depending on their size and deformability, density, and electric charge. Size‐based CTC isolation relies on microfabricated filters, such as 2D filter membranes (e.g., ISET, CellSieve, and ScreenCell), and 3D microfilters (FaCTChecker and Parsortix), to isolate single CTCs or CTC clusters based on their size and/or deformability [67]. Although CTCs (12–25 µm) are typically larger than WBCs (8–14 µm), false‐positive results might be obtained due to variations in CTC size as a result of apoptosis, clustering, or cell cycle stage [67]. Integration with flow cytometry was sought in some trials to improve the size‐based sorting of CTCs [68].

CTCs can also be isolated using hydrodynamic microfluidic devices that depend on inertial focusing to separate CTCs from other blood cells, exploiting centrifugal, Coriolis, and Euler forces to transport and manipulate liquids through their interaction with microstructures [69]. The term, inertial focusing, refers to migration of cells across streamlines into equilibrium positions within the flow cross section as they migrate downstream in a microchannel. However, the purity of CTCs is usually low due to the size overlap between CTCs and WBCs. Also, cell–cell interactions might affect the cell‐focusing behavior and reduce the separation efficiency. Recently, an inertial focusing based labyrinth device was demonstrated to be capable of isolating single CTCs and CTC clusters from the blood of NSCLC patients with 100% and 96% cell recovery, respectively [70]. Other inertial focusing platforms that can isolate viable CTCs have also been introduced. The ClearCell Fx platform is an automated hydrodynamic centrifugation system that employs Dean flow fractionation (DFF) to separate between viable CTCs and other blood cells (Fig. 2E) [71]. The Vortex method hydrodynamically traps CTCs in side channels at high flow rates (Fig. 2F) [72].

Density‐based CTC isolation technologies (e.g., Ficoll gradient centrifugation), employ gradient centrifugation to isolate CTCs from other hematopoietic components according to their sedimentation rate. CTCs and mononuclear cells have lower buoyant densities (< 1.077 g·mL^−1^) unlike RBCs and neutrophils (> 1.077 g·mL^−1^) and can thus be extracted from the top layer. The advantage of this method is that it offers a simple way to isolate CTCs. However, the sensitivity of this system is poor due to the leakage of CTCs to the plasma layer or the formation of large CTC aggregates settling to the bottom of the gradient. This problem was solved using the OncoQuick gradient system (Greiner Bio‐One, Frickenhausen, Germany), which includes a porous barrier above the density gradient to prevent mixing with the whole blood [65]. This system has been demonstrated to achieve higher CTC enrichment compared to the standard Ficoll gradient method.

Electric charge‐based approaches exploit dielectrophoresis (DEP) to isolate CTCs based on their distinct dielectric properties, leading different dielectrophoretic migration compared to other cell types [73]. DEP separation techniques can achieve a single‐cell level purification. However, the process is slow because the separation requires a low fluid velocity, resulting in low sample throughput. A commercially available DEPArray technology combining the precision of microfluidics and power of microelectronics, has been utilized to isolate CTCs from breast and colon cancer patients [74, 75].

#### 3.2.3 Combined approaches

The choice of a particular CTC enrichment technology is driven primarily by the analyte to be measured. For example, molecular profiling of CTCs would require technologies capable of isolating pure CTCs to improve the quality of molecular information gleaned from the assay. Eliminating the need for sample preprocessing prior to CTC separation is also crucial to avoid cross‐contamination and reduce sample loss. Antigen‐independent CTC enrichment approaches entail less complicated workflows, but the CTC purity is typically low. Antigen‐driven approaches permit the isolation of defined CTC phenotypes, making them compatible with molecular assays. However, these approaches require highly specific antibodies or even antibody cocktails as well as a robust release strategy.

Both antigen‐dependent and antigen‐independent CTC enrichment approaches have their own advantages and limitations and hence combining both methods has been pursued in an attempt to improve overall CTC purity and recovery. For instance, using a geometrically enhanced differential immunocapture (GEDI) chip, which sorts CTCs based on their size and immunoaffinity properties, can improve cell recovery, yielding CTC counts much higher than the CellSearch system [76]. Also, the CTC‐iChip can isolate CTCs from large volumes of blood by micropillar arrays, hydrodynamic size‐based sorting, and magnetophoresis, permitting the recovery of pure cells which are suitable for cell culture and downstream profiling (Fig. 2G) [77].

It is worth noting that for CTC analysis at the single‐cell level, CTC enrichment might be combined with another isolation step using fluorescence‐activated cell sorting (FACS) [78], laser microdissection [79], or manual [80] and automated micromanipulation [81] to isolate single CTCs.

### 3.3 Molecular profiling of CTCs

In addition to CTC enrichment, comprehensive molecular and functional profiling of isolated CTCs is highly desirable to characterize their metastatic potential and resistance to therapy [82].

#### 3.3.1 Genetic analysis of CTCs

Quantitative real‐time polymerase chain reaction (qRT‐PCR) utilizes primers designed to target specific genes of interest and has been used to investigate the transcriptional landscape of CTCs from different types of cancer [48]. However, the method is susceptible to amplification bias and is unable to distinguish between viable and apoptotic cells [82]. There is also a significant loss of mRNA molecules during purification and processing steps.

Fluorescence *in situ* hybridization (FISH) is a cytogenetic technique that uses fluorescent probes to specifically detect the presence of specific DNA or RNA sequences in individual cells [83]. FISH might require labeling with multiple fluorescent probes to attain a sufficient signal, which might preclude accurate quantification. However, a quantifiable, dual‐colorimetric RNA‐*in situ* hybridization (ISH) technique was introduced to characterize EMT in CTCs collected from breast cancer patients [26].

Comparative genomic hybridization (CGH) is a cytogenetic method for analyzing copy number variations (CNVs) relative to ploidy level in the DNA of a test sample compared to a reference sample. Each DNA sample is labeled with different fluorescent molecules and the difference in fluorescence ratio is used to determine the DNA copy number along the chromosome [84]. CGH is used to detect unbalanced chromosomal changes and structural rearrangements inside the cell. A more specific form of CGH based on the use of DNA array, thus referred to as (ACGH), has been used to reveal copy number aberrations among single CTCs collected from patients with metastatic colorectal cancer [85].

Next‐generation sequencing is used for in‐depth genomic analysis of CTCs. Single‐cell RNA sequencing includes reverse transcription, cDNA amplification, and preparation of a sequencing library. After transcription, minute amounts of cDNA might be amplified, leading to amplification biases [86]. Third‐generation sequencing methods alleviated this constraint, allowing for sequencing of nonamplified cDNAs, which is tremendously valuable for CTC analysis at the single‐cell level [87]. This powerful approach has been used to evaluate the inter‐ and intra‐tumoral heterogeneity (ITH) in CTCs, showing that ITH is a result of clonal evolution and presence of cancer stem cells [88]. Also, it was demonstrated that CTCs retrieved from prostate cancer patients shared 90% and 70% of the mutations present in the primary tumor and metastatic sites [89]. This study suggested that CTCs might originate from both the primary tumor and its metastatic lesions.

#### 3.3.2 Protein‐level analysis for CTCs

Immunostaining is the most popular approach for analyzing proteins in CTCs. For instance, epithelial CTCs are commonly identified based on the presence of specific proteins, such as EpCAM, CK proteins, and absence of CD45 [44]. In addition, immunostaining for more biomarkers has been used for identification of CTCs in specific types of cancer, such as EGFR in prostate cancer and HER2 in breast cancer. The method is somewhat limited by the availability of specific antibodies. Several single‐cell approaches have been developed to analyze proteins in CTCs, such as MagRC [90], single‐cell barcode chip (SCBC) [91], and single‐cell western blotting (scWestern) [92].

#### 3.3.3 Functional analysis of CTCs

The invasion assay is used to evaluate the invasiveness of CTCs based on their ability to digest a fluorescently labeled adhesion matrix. For example, this assay was used in conjunction with the MagRC platform to investigate the invasiveness of CTCs with low EpCAM expression. This study revealed the dynamic heterogeneity of CTCs and their phenotypic changes by mimicking the multistep metastatic cascade [93].

The EPISPOT assay is used to analyze tumor‐specific proteins released by CTCs [94]. In this assay, the cells are seeded and cultured in EPISPOT plate, which contains a nitrocellulose membrane coated with an antibody specific to the secreted protein of interest. During an incubation period (24–48 h), the secreted protein is captured by the immobilized antibody. The cells are then washed off and the secreted protein is detected using a fluorescently labeled secondary antibody. The fluorescent immunospots are then counted and used to estimate the number of viable CTCs that actively secrete proteins. The EPISPOT assay was used to demonstrate that viable epithelial tumor cells secrete full‐length CK19 and that CK19‐secreting cells might constitute a highly metastatic subset of breast cancer cells [95].

### 3.4 Monitoring drug resistance and informing drug selection

Currently, selection of targeted therapy in patients with metastatic cancer is solely based on the analysis of a primary tumor. A metastatic relapse might be encountered many years after tumor resection, while the data available from the resected primary tumor could be outdated. On the contrary, CTC analysis offers a means for interrogating the tumor repeatedly over the course of treatment. Evidence that CTCs can be used as a surrogate for the tumor tissue to monitor drug resistance came from two main observations. First, implantation of CTCs collected from patients into immunodeficient mice led to the formation of CTC‐derived explant models that recapitulate the donor's response to chemotherapy [37]. Second, molecular analysis of individual CTCs showed distinct somatic gene copy‐number profiles in patients with chemosensitive and chemorefractory SCLC [96]. In the following sections, we focus on how CTC analysis can contribute to elucidation of therapeutic resistance and effective personalized cancer therapy.

#### 3.4.1 CTC count and therapeutic response

The CellSearch system has been used in several studies to investigate whether CTC enumeration carried out at baseline and during chemotherapy might be predictive of patient's response to treatment. In 2008, a landmark article demonstrated the prognostic value of CTC count in patients with metastatic castration‐resistant prostate cancer (mCRPC) when conducted before initiating a new treatment [97]. Although the baseline CTC count did not appear to be predictive of treatment response, the change in CTC count during treatment has offered a reliable marker of response to treatment in different clinical settings. In the IMMC38 study, mCRPC patients with high baseline CTC counts that changed to low counts after chemotherapy had better clinical outcomes than those patients whose baseline CTC counts remained high even after treatment [97]. These findings were later confirmed by a randomized phase III study that led to the approval of the prostate cancer drug, abiraterone [98]. Similarly, in metastatic breast cancer patients, a drop in CTC count after 4 weeks of treatment with first‐line therapy correlated with improved survival [99]. The prognostic value of CTC count has also been established in other types of cancer, such as lung and colorectal cancers. A clinical study revealed that metastatic colorectal cancer patients with low CTC counts after therapy had extended progression‐free and overall survival [100]. Also, the reduction of CTC count in SCLC patients after several cycles of chemotherapy was found to be a strong predictor of overall survival [101].

Variations in CTC count after drug exposure can also be used to study the pharmacodynamics of new drugs during early‐phase clinical trials. For example, a phase I trial of the PARP inhibitor, niraparib, has exploited changes in CTC count and nuclear γH2AX expression as endpoints of antitumor activity on a cohort of mCRPC patients [102]. A phase I study of ARQ197, a selective inhibitor of the hepatocyte growth factor receptor c‐MET, evaluated the changes in CTC count in patients with different tumors [103]. A phase I trial of EZN‐4176, a second‐generation antisense oligonucleotide to exon 4 of the androgen receptor, has utilized the variations in CTC count to evaluate the drug efficacy in prostate cancer patients [104].

**Fig. 3:**
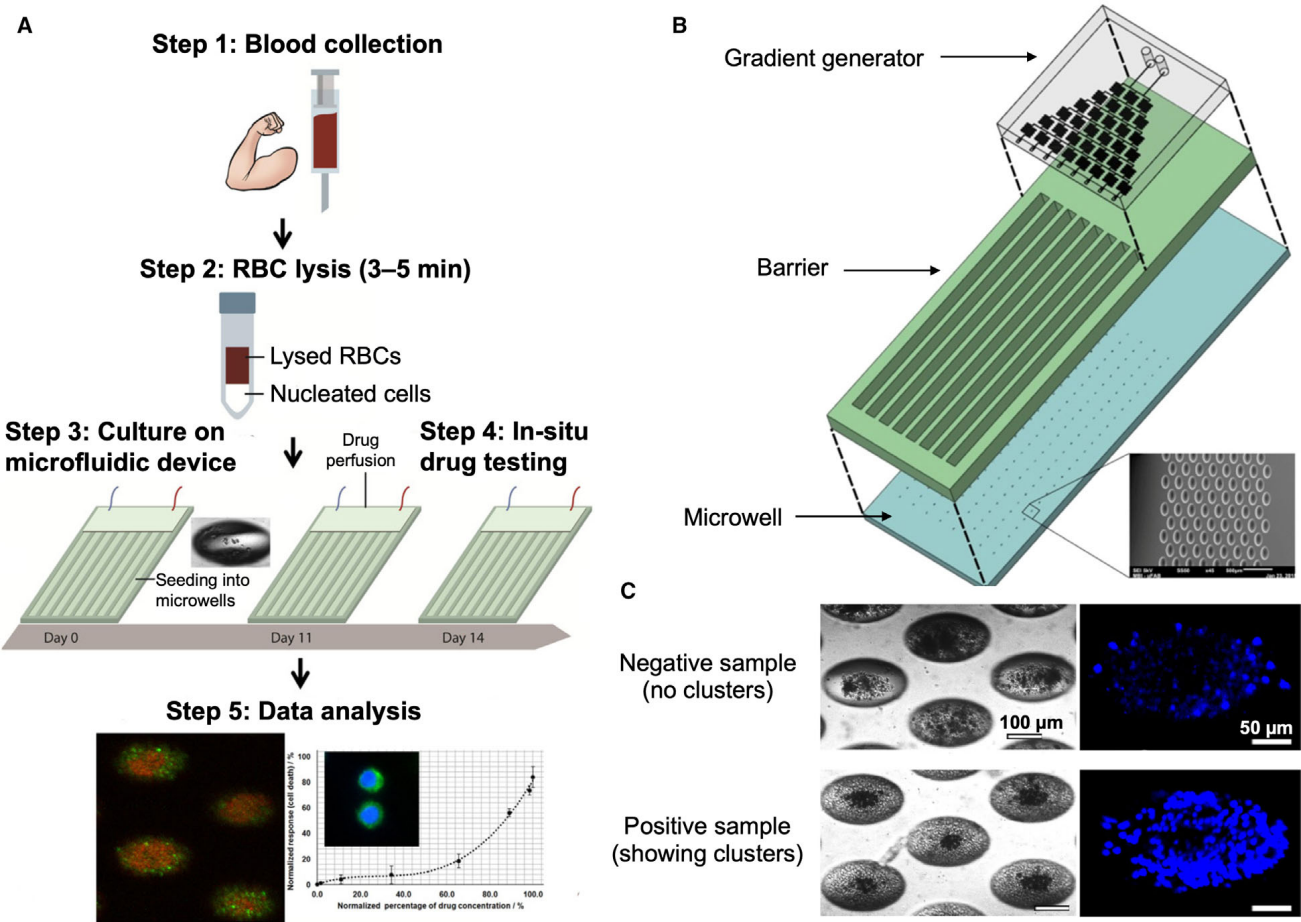
CTC cluster assay for drug screening. (A) Schematic depicting the cluster assay for anticancer drug screening. Blood samples are first lysed to remove red blood cells (RBCs), and the nucleated cell fraction is then seeded into an integrated microwell‐based microfluidic device. Drugs are introduced directly *in situ*, and the CTC clusters are generated within 2 weeks. (B) Three‐dimensional layout of the drug assay device showing the gradient generator, barrier, and microwells. (C) Representative bright‐field and fluorescent images of negative and positive samples. Nuclei were stained using Hoechst dye. Figures adapted with permission from Ref. [105].

The relationship between the number of CTC clusters and drug resistance was also investigated [105]. CTCs were collected from patients with early‐stage, locally advanced, or refractory metastatic breast cancer. Patient‐derived CTCs were cultured in an integrated microfluidic system incorporating tapered microfabricated wells for drug testing *in situ* (Fig. 3A,B). It was found that cluster formation correlated inversely with increased drug concentration (Fig. 3C). The method permitted determination of the median inhibitory concentrations after two weeks of treatment, allowing for rapid intervention upon detection of drug resistance or tolerance.

Although several researchers have independently confirmed the prognostic value of CTC count alterations, no consensus has been reached on the optimal cutoff threshold for clinical prognostication [106]. Several factors are likely to contribute to this problem. First, several CTC detection technologies with different cutoffs for CTC enumeration have been used in different clinical studies, which made it difficult to compare the results and draw firm conclusions. Second, CTC isolation methods that target epithelial markers, such as EpCAM, might underestimate the CTC count in metastatic diseases [107]. Third, the lack of studies showing that switching of treatment in patients with persistent CTCs might have an effect on either progression‐free or overall survival. For example, a phase III clinical trial by the Southwest Oncology Group (SWOG S0500 study) found that changing therapy according to CellSearch results did not affect either progression‐free or overall survival of cancer patients [108]. For these reasons, studies that investigated the prognostic value of CTC count are relatively old and no new studies have been pursued. However, molecular characterization of CTCs at the genotypic and phenotypic levels has the potential to identify predictive biomarkers of response to personalized therapies.

#### 3.4.2 Genomic aberrations and resistance

CTCs provide a valuable source for studying the genomic aberrations in cancer patients. Genomic‐level assays can be used to analyze CTCs for the presence or absence of key signaling oncogenic mutations. However, the driver mutation landscape usually changes as a result of the outgrowth of drug‐resistant subclones after exposure to systemic therapy. It is thus necessary to assess the molecular indicators of resistance in CTCs at the time of starting new line of targeted therapy and repeatedly over the course of treatment to guide clinicians on when to stop or change the treatment plan. As a case in point, a study on 254 breast cancer patients revealed the presence of HER2 overexpression in almost one‐third of the patients who had no HER2 overexpression in the primary tumor [109]. This finding had a remarkable clinical impact as overexpression of HER2 is a biomarker of response to HER2‐targeting drugs in breast cancer [110]. Another study revealed the presence of activating mutations in the EGFR‐encoding gene in CTCs collected from lung cancer patients receiving EGFR‐targeting tyrosine kinase inhibitors (TKIs). Interestingly, some of these mutations were absent in the primary tumor, indicating the de novo emergence of these mutations [111]. Both examples highlighted the superior predictive value of CTC assays compared to conventional tissue biopsies.

EGFR mutations can affect the outcome of EGFR TKIs in lung cancer patients. In one study, CTCs were isolated from NSCLC patients using the CTC‐chip and *EGFR* mutations were analyzed using the Scorpion Amplification Refractory Mutation System (SARMS) [111]. Analysis of CTCs isolated from *EGFR*‐mutant patients demonstrated a decrease in CTC count and improved radiological response within a week after starting a course of treatment with the EGFR TKI, gefitinib. In addition, analysis of CTCs obtained from patients with increased CTC count after treatment revealed the presence of *EGFR* T790M mutation, which was found responsible of resistance to EGFR TKIs. In a subsequent study, The CTC‐Chip was used to isolate the CTCs from NSCLC patients recurring after treatment with first‐generation EGFR TKIs [112]. The CTCs isolated from 76% of the patients were genotyped and the *EGFR* T790M mutation was identified in 50% of the cases. Interestingly, the mutational status of CTCs and primary tumors was concordant in only 57% of the patients, providing further evidence that molecular profiling of CTCs would better reflect the heterogeneous nature of the tumor. The emergence of *EGFR* T790M mutation in CTCs isolated from NSCLC patients treated with EGFR TKIs was also demonstrated using a different approach for CTC enrichment based on gradient centrifugation and negative enrichment prior to molecular characterization with droplet digital PCR [113]. Another study assessed the predictive value of CTCs harboring *EGFR* DelEx19 mutation in NSCLC patients [114]. The CTCs were isolated using gradient centrifugation and molecularly analyzed with qRT‐PCR. It was noted that 50% of the patients receiving EGFR TKIs were responsive to treatment and turned negative for *EGFR*‐mutant CTCs.

Mutations in KRAS—a protein downstream of EGFR—block the efficacy of EGFR‐targeting therapy. Meta‐analysis of the mutational status of *KRAS* in CTCs and tumor tissues revealed discrepancies in colorectal cancer patients [115]. Isolation of CTCs from colorectal cancer patients using the CellSearch system followed by their comprehensive genetic analysis with ACGH revealed mutations in known driver genes, such as *KRAS*, *APC*, and *PIK3CA* [85]. These mutations were also observed in the primary and metastatic tumors of the same patients. Remarkably, high level of amplification of *CDK8*, a potential target for CDK inhibitors, was observed in some of the isolated CTCs.

*BRAF* mutations have been utilized as predictors of response to targeted therapies in melanoma and colorectal cancer. For example, CTCs were detected in smaller numbers in patients with metastatic BRAF^v600E^ melanoma and declined after BRAF‐targeted therapy [116]. In this study, CTCs were isolated using the ^HB^CTC‐Chip; however, the low purity of CTCs precluded further molecular analysis. In a following study, the molecular signature of melanoma‐derived CTCs was revealed [117]. CTCs were isolated from metastatic BRAF^v600E^ melanoma patients using the CTC‐iChip. A panel of 19 specific RNA transcripts was quantitatively analyzed with digital PCR, providing a CTC score. A decrease in CTC score within 7 weeks of treatment with immune checkpoint inhibitors was found to correlate with improvement in progression‐free and overall survival.

In breast cancer, resistance to HER2‐targeting therapy can occur as a result of *PIK3CA* mutations, which has been reported in 15.9% of breast cancer patients [118]. However, *PIK3CA* mutations were detected more frequently in metastatic breast cancer patients with HER2‐negative tumors and HER2‐positive CTCs [119]. Also, *ESR1* mutations have emerged as acquired endocrine resistance mechanism in estrogen receptor (ER)‐positive metastatic breast cancer patients [120]. In a proof‐of‐concept study, CTCs were isolated from ER‐positive breast cancer patients using the CTC‐iChip. These CTCs were used to generate CTC lines, which were screened for mutations in a panel of 1000 annotated cancer genes using next‐generation sequencing [121]. Sequencing results revealed mutations in *PIK3CA*, *ESR1*, and *FGFR2* genes, among others. Drug sensitivity testing of one CTC line harboring activating mutations in *PIK3CA* and *FGFR2* revealed responsiveness to PIK3CA and FGFR2 inhibitors, whereas combined inhibition exhibited a cooperative effect.

Mutations in the androgen receptor (*AR*) gene were identified in CTCs from CRPC patients. Amplification of *AR* leads to overexpression of AR protein and hypersensitization of prostate cancer cells to residual androgens in patients receiving drug‐induced castration therapy. Mutations in the *AR* gene were associated with resistance to hormone therapy and blockade therapy was found to improve the survival rate of CRPC patients [122]. A protocol has been developed to combine CTC enumeration with the CellSearch system and characterization of *AR* amplification by FISH in an integrated CellSearch cartridge [123]. Using this protocol, *AR* amplifications were readily observed in CRPC patients. Interestingly, marked heterogeneity in *AR* gene copy numbers was observed in CTCs collected from the same patient. A similar observation was reported when ACGH analysis was carried out on CTCs isolated by FACS and immunomagnetic enrichment [124].

The most significant predictive value of CTCs has been highlighted by several reports demonstrating the correlation between the existence of *AR* splice variant 7 (AR‐V7)—coding for a truncated and constitutively active AR—and resistance to anti‐AR therapy [125, 126]. A case in point is a clinical study on two groups of CRPC patients receiving either abiraterone or enzalutamide [126]. Using the AdnaTest platform, the CTCs were isolated and AR‐V7 was detected with PCR. None of the patients harboring AR‐V7*^pos^* CTCs had a 50% PSA response rate compared to 68% and 53% of the patients with AR‐V7*^neg^* CTCs and progressing on abiraterone or enzalutamide, respectively. Using the same methodology, the presence of AR‐V7*^pos^* CTCs was observed in 46% of CRPC patients receiving taxanes [125]. However, the PSA response rate did not vary significantly between the AR‐V7*^pos^* and AR‐V7*^neg^* patients. Also, the progression‐free survival of AR‐V7*^pos^* patients treated with taxanes was longer than the patients treated with abiraterone or enzalutamide from the previous study. Admittedly, this indirect comparison between patients from different cohorts has to be interpreted more carefully, particularly because patients treated with taxanes had more advanced disease.

The AR splice variants 1, 3, 4, 7, and 12 were also analyzed by RNA sequencing of single CTCs isolated using the CTC‐iChip followed by cell picking by a micromanipulator [127]. This study revealed heterogeneous expression levels of the different splice variants between and within prostate cancer patients progressing on an AR inhibitor. These splice variants were not detected in the primary tumors, indicating that alternative splicing occurs during disease progression. AR‐V7 was the most frequently formed variant in 36% of the CTCs and 73% of the patients. Retrospective analysis of CTCs indicated the activation of noncanonical Wnt signaling and that heterogeneity in signaling pathways among single CTCs might have contributed to treatment failure. Analysis of AR‐V7 in CTCs isolated from prostate cancer patients has also been demonstrated using the MagRC platform [55]. Compared to other microfluidic platforms, the MagRC platform enabled the isolation of CTCs and analysis of specific mRNAs in one step and did not require any genotyping modalities. Instead, mRNA expression is determined by enumerating magnetically ranked CTCs and their distribution in the MagRC device, without interference from nonspecifically captured hematopoietic cells.

In addition to *AR*, *TMPRSS2‐ERG* rearrangements, resulting from fusion of an *ERG* oncogene and AR‐driven *TMPRSS2* promoter, have been detected in more than 50% of prostate cancer patients [128]. The finding that patients with specific *ERG* rearrangements were more sensitive to anti‐AR therapy has shed light into the potential predictive value of these rearrangements and called for further trials [129]. In this study, CTCs were isolated from prostate cancer patients using the CellSearch system and characterized by FISH. Patients harboring *TMPRSS2:ERG* rearrangements responded better to abiraterone in terms of PSA response than patients in whom rearrangements were absent. However, the results shown by another study could not confirm such correlation [130]. The CellSearch system was also used for CTC enrichment from mCRPC patients, but the expression of TMRSS2:ERG was determined with qRT‐PCR. Few years later, the potential role of *TMPRSS2:ERG* as predictive marker to taxane therapy was confirmed [131]. However, further studies are still needed to clarify the discordance between the results and allow for prospective evaluation of the predictive value of *TMPRSS2:ERG* rearrangements.

Some studies assessed the possibility of using CTCs as a surrogate to detect other genomic rearrangements, such as *ALK* and *ROS1*, in lung cancer patients. In one study, CTCs were isolated from NSCLC patients using the ClearCell Fx system and *ALK* rearrangement patterns were detected with FISH [132]. It was noted that *ALK^pos^* CTC counts were higher in *ALK^pos^* patients compared to *ALK^neg^* patients and healthy donors. In addition, the study revealed an association between the count of *ALK^pos^* CTCs and response to the ALK inhibitor, crizotinib. Furthermore, a copy number gain (CNG) of oncogenic *ALK* in CTCs was found to contribute to disease progression despite treatment with crizotinib. In this respect, *ALK* CNG has also been hypothesized as a possible mechanism of resistance to ALK inhibitors in other studies [133, 134]. Using a different enrichment approach, the NanoVelcro chip was used to isolate CTCs from NSCLC patients and *ALK* rearrangements were detected with FISH. A correlation between the number of *ALK^pos^* CTCs and disease progression was demonstrated in patients treated with crizotinib [135].

The ISET filtration system and the filter adapted (FA)‐FISH method were used to detect *ROS1* rearrangements in CTCs obtained from four NSCLC patients treated with crizotinib [136]. In this study, *ROS1^pos^* CTCs were detected in all patients with *ROS1* rearrangement previously detected by a tumor biopsy. The number of *ROS1^pos^* CTCs was reduced in two patients who responded to crizotinib treatment, whereas no change was observed in one nonresponsive patient to crizotinib. In addition, the *ROS1* CNG was assessed and it was found that number of *ROS1* copies in *ROS1^pos^* CTCs had increased in two patients experiencing disease progression despite treatment.

#### 3.4.3 Altered proteins and resistance

Surface proteins of CTCs have been established as suitable biomarkers for monitoring tumor resistance while targeted therapies directed toward these proteins have been approved in several types of cancer, such as breast, prostate, and lung cancers. Also, several single‐cell assays have been developed to analyze proteins in CTCs, despite their low yield in most cancer patients [41, 137]. In breast cancer, some of the most successful therapeutic agents are directed toward ER and HER2. Breast cancer patients with ER‐positive primary tumors can harbor ER‐negative CTCs [33]. Also, HER2‐positive CTCs were detected in patients with HER2‐negative primary tumors [109]. The discordance between ER or HER2 expression in CTCs and primary tumors was suggested as a potential cause of breast cancer resistance to endocrine or targeted therapy, respectively.

In one study, CTCs were isolated from metastatic breast cancer patients using the CellSearch system and two markers of endocrine sensitivity (ER and BCL2) were analyzed by immunostaining [138]. The study demonstrated a decrease in the number of ER^pos^ CTCs during treatment with the selective ER downregulator (SERD), fulvestrant. The data also suggested a correlation between ER and BCL2 expression in CTCs, which supported the notion that ER^neg^ CTCs might lack ER signaling.

Two multicenter studies have evaluated the predictive value of HER2^pos^ CTCs in patients with HER2^neg^ breast cancer and revealed higher survival under HER2‐targeted therapy with trastuzumab [139, 140]. A third study showed that the presence of HER2^pos^ CTCs was not associated with tumor size, hormone receptor status, axillary lymph node involvement or histopathological grading [141]. In these studies, the CTCs were isolated using the CellSearch System and HER2 was detected with immunostaining. Besides other findings [142, 143], these reports suggested the need for determining the HER2 status in CTCs from higher number of patients in clinical trials to assess the efficacy of HER2‐targeted therapy.

PD‐1/PD‐L1 expression has been recognized as a suitable predictive biomarker for monitoring tumor response to checkpoint blockade immunotherapy, which has been approved in several types of cancer, such as NSCLC, breast, gastrointestinal, and head and neck cancer [144]. A correlation between PD‐L1 expression in CTCs and primary tumors was reported [145]. The expression of PD‐L1 in CTCs has been utilized as a biomarker for response to PD‐1 checkpoint inhibitors, such as nivolumab and pembrolizumab. In one study, the CellSearch system was used to isolate the CTCs from NSCLC patients treated with nivolumab and PD‐L1 positivity was detected with immunostaining [146]. After 6 months of treatment, patients with PD‐L1^neg^ CTCs were found responsive to therapy, whereas the persistence of PD‐L1^pos^ CTCs was associated with lack of response. Another study evaluated the expression of PD‐1/PD‐L1 in ISET filtration‐enriched CTCs isolated from NSCLC patients before and after chemotherapy [147]. The results showed a decrease in the number of PD‐1^pos^ CTCs associated with an increase in the number of PD‐L1^pos^ CTCs after three cycles of chemotherapy, suggesting that chemotherapy might have eliminated PD‐1^pos^ CTCs. Several reports have confirmed the presence of PD‐L1^pos^ CTCs in NSCLC patients using different enrichment technologies, such as the Vortex method and ClearCell FX system [148, 149]. In addition to qualitative PD‐L1 detection, one study revealed a correlation between the abundance of PD‐L1 in CTCs and the response to PD‐1/PD‐L1 blockade therapy in patients with advanced gastrointestinal tumors [150]. Immunofluorescence was used to quantify PD‐L1 expression in magnetically enriched CTCs. Prior to treatment, the abundance of PD‐L1 in CTCs was found to serve as a predictor of the therapeutic response.

Although the majority of protein assays have been applied to cell‐surface proteins, analysis of intracellular proteins is particularly beneficial for assessing the tumor response to therapy because intracellular oncoproteins are responsible of the initiation and progression of tumors [151]. For example, the subcellular localization of AR has been used as biomarker of response to abiraterone, enzalutamide, and taxanes. After activation by androgens, AR is translocated from the cytoplasm to the nucleus to act as a transcription factor for target genes. Thus, the nuclear localization of AR in tumor cells indicates an active AR signaling status [152]. In one study, it was demonstrated that the absence of nuclear AR in mCRPC patients treated with docetaxel correlated with clinical response, as assessed by immunostaining of AR in CTCs enriched using the CellSearch system [153]. This was ascribed to the ability of taxanes to inhibit ligand‐induced AR nuclear translocation and downstream transcriptional activation of AR target genes, such as PSA. Overall, 71% of the patients who responded to taxanes had cytoplasmic AR, whereas 72% of the CTCs sampled from patients with disease progression have exhibited nuclear AR. In another report, the AR abundance in CTCs obtained from CRPC patients progressing on abiraterone was determined using the ImageStreamX system, which is based on microscopy and flow cytometry [154]. The median AR staining intensity was found to be three times higher in patients progressing on abiraterone compared to patients who were abiraterone‐naïve, whereas no difference in AR subcellular localization was observed. AR signaling was studied by others to investigate the response of mCRPC patients to ADT [155]. In one study, CTCs were captured using the CTC‐Chip and analyzed with immunostaining for PSA and its membrane‐bound form, PSMA. Accordingly, the captured CTCs were assigned into three different categories with respect to AR signaling, including AR‐off (PSA^neg^/PSMA^pos^), AR‐mixed (PSA^pos^/PSMA^pos^), and AR‐on (PSA^pos^/PSMA^neg^). Initiation of first‐line ADT induced a marked phenotypic switch from AR‐on to AR‐off CTCs, followed by the disappearance of CTCs after three months. By contrast, secondary hormonal therapy resulted in a variable response. In addition, the presence of AR‐on and AR‐mixed CTCs in patients treated with abiraterone was associated with adverse clinical outcome. Interestingly, a correlation between AR activation and upregulation of PSA and PSMA was observed, which made it possible to predict the outcome of AR‐based therapy based on PSA and PSMA levels.

**Fig. 4:**
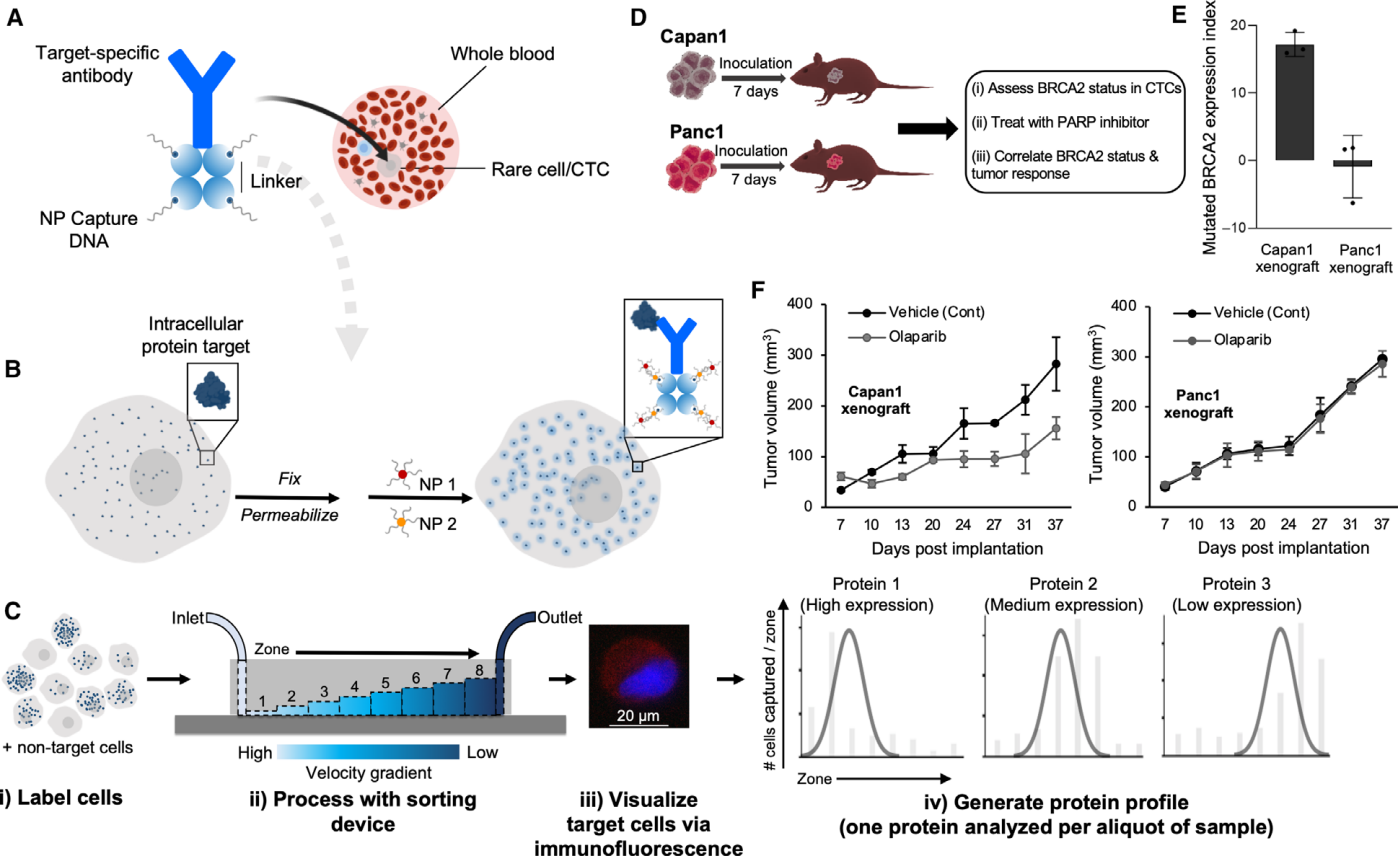
Analysis of intracellular proteins using the MagRC approach. (A) An antibody specific for the target intracellular protein is tagged with streptavidin then modified with biotin‐labeled ssDNAs using biotin–streptavidin coupling. (B) The cells expressing the target intracellular protein are fixed, permeabilized, and incubated with the intracellular protein‐specific antibody modified with ssDNAs. The ssDNAs are then hybridized with two capture probes (CP1 and CP2), which are composed of complementary DNA sequences modified at one end with MNPs. Aggregates of MNPs are thus formed and trapped within the cells that express the intracellular protein. (C) The cells are sorted using a microfluidic device featuring eight capture zones, immunostained, and counted to generate a profile characteristic for the target protein. (D) Schematic illustration for therapeutic protein analysis in xenografted mice. (E) Analysis of mutated BRCA2 protein in CTCs captured from the blood of mice bearing either Capan1 (with mutated BRCA2) or Panc1 (with wild‐type BRCA2) xenograft. The analysis was carried out at day 7 after tumor formation. After tumor formation, the mice were randomly divided into control and treated groups. Mice in the treated group received 50 mg·kg^−1^ olaparib, whereas mice in the control group received only the vehicle. (F) Tumor volume is plotted against duration of treatment for Capan1 and Panc1 xenografts. Figure reprinted with permission from Ref. [54].

Unlike intracellular proteins that are expressed by tumor cells as well as normal cells, genetically altered oncoproteins are produced only by tumor cells and thus proteins harboring tumor‐specific fusions or mutations have become targets to molecular therapeutics. For example, the mutated BRCA2 protein has been utilized as a predictive biomarker in patients with pancreatic, breast, or ovarian cancer [156]. BRAC2 mutations result in impaired homology‐directed DNA repair, making these tumors sensitive to DNA repair‐targeting drugs, such as PARP inhibitors [157]. In 2018, the FDA approved the PARP inhibitor, olaparib, for treatment of patients with advanced breast and ovarian cancers harboring BRCA mutations [158]. More recently, the MagRC platform was featured as a technology capable of analyzing intracellular proteins in the nucleus, mitochondria and cytoplasm of rare tumor cells, without interference from hematopoietic cells [54]. The approach relied on the use of magnetic amplification reagents to facilitate sensitive detection of low‐abundance proteins (Fig. 4A,B). After labeling the target protein with the magnetic reagents, the protein‐harboring cells were magnetically ranked using a MagRC device featuring eight capture zones. The number of captured cells as well as their distribution among the capture zones were used to determine the protein level (Fig. 4C). This approach was proven capable of tracking the expression of a variety of therapeutic proteins in CTCs isolated from orthotopic prostate cancer xenografts as well as from mCRPC patients. Remarkably, the method was used to detect mutated BRCA2 protein in CTCs isolated from pancreatic tumor xenografts (Fig. 4D) and predict how mice with drug‐sensitive and drug‐resistant tumors would respond to the PARP inhibitor, olaparib (Fig. 4E).

## 4 CTC analysis in clinical care

Regulation of tumor biomarker tests is a complex process and even tests approved by the FDA may not have established clinical utility [159]. Nevertheless, there are many commercially available tests that have not been submitted to the FDA for approval but are instead marketed as laboratory‐developed tests (LDTs). Although LDTs must be certified, these tests do not follow the same guidelines applied by the FDA to other diagnostics and their analytical and/or clinical validity might not be thoroughly reviewed [159]. The semi‐automated CellSearch platform (Menarini Silicon Biosystems Inc., Huntington Valley, PA, USA), while not a microfluidic technology, is the only FDA‐cleared method for CTC detection in patients with metastatic breast, prostate, or colorectal cancer [43]. The analytical and clinical validity of CTC enumeration have been verified for prognostication of patients with metastatic breast, prostate, or colorectal cancer, as well as patients with nonmetastatic breast cancer treated with chemotherapy [160]. Clinical studies have demonstrated reduced progression‐free and overall survival for patients with metastatic breast, prostate, and colorectal cancer harboring ≥ 5 CTCs, ≥ 5 CTCs, or ≥ 3 CTCs per 7.5 mL blood, respectively [97, 99, 100]. In other cancers, such as pancreatic [161] and ovarian [162], the CTC counts were lower, and the test has not been approved by FDA.

Although more than a decade has passed since its FDA approval, the CellSearch platform has not been widely adopted in recent tumor resistance studies due to the reports showing that patients with high CTC counts might not benefit from switching treatment plans [163]. In addition, the recovery of CTCs by the CellSearch system is relatively low compared to other technologies that can recover orders of magnitude higher CTC counts from smaller sample volumes [90]. Furthermore, the CellSearch platform cannot be utilized to monitor mesenchymal CTCs with downregulated EpCAM and/or CK expression, which have been implicated in therapeutic resistance [26]. Notably, the CellSearch platform could only detect CTCs in the blood of only 32% of 101 metastatic NSCLC patients [164].

In addition, there is a FDA‐approved test for the constitutively active AR‐V7 associated with prostate cancer resistance to ADT using a commercially available platform from Epic Sciences [165]. There are also several ongoing clinical trials in which therapeutic decisions are made according to the molecular or phenotypic characteristics of CTCs obtained from different types of cancer, such as DETECT‐IV in breast cancer and CABA‐V7 in prostate cancer [6]. Also, several platforms for antigen‐independent CTC enrichment are currently under evaluation but are not yet approved for clinical practice [9]. The ‘no cell left behind’ platform from Epic Sciences can capture all nucleated cells from a clinical specimen on slides. The captured cells are then analyzed with multiplexed immunofluorescence assays using proprietary algorithms to identify CTCs, followed by physical picking of single cells for further downstream analysis [166].

## 5 Conclusions

CTC‐based liquid biopsy is currently entering a third wave of clinical development, following the establishment of laboratory platforms and promising results from evaluative studies. CTCs have been shown to preserve the molecular identity of primary tumors while yielding a renewable source of informative material. Profiling CTCs may provide early surrogate endpoints for clinical outcomes, thereby reducing the long follow‐up periods and substantial costs associated with clinical trials on neoadjuvant and/or adjuvant therapies. CTCs are also amenable to rapid phenotypic and functional readouts, that when coupled with iterative computational approaches, will contribute to a systematic understanding of the mechanisms of tumor progression and therapeutic resistance.

One of the key advantages of using CTCs for liquid biopsy is that this approach offers an opportunity to perform a range of analyses at the DNA, RNA, and protein level, as well as permitting functional studies. Extending the range of information extractable from CTCs by interrogating multiple analytes would likely improve their diagnostic power in terms of both specificity and sensitivity. In addition, multidimensional assays of several liquid biopsy markers would provide a more effective means to predicting tumor response to therapy. For instance, ctDNA‐based tests are more sensitive than CTC assays in identifying gene mutations in cancer patients [167] and can be used to detect minimal residual disease (MRD) or clinical relapse in an early stage [168]. As complementary tools, interrogation of CTCs and ctDNA allowed for robust monitoring tumor response to targeted therapy in NSCLC patients [169]. Integrative analysis of other liquid biopsy markers such cfRNAs and EVs is foreseen to advance the field of precision oncology in the near future, whereas other candidates such as TEPs and tumor metabolites are likely to contribute in the longer term.

Developments in assay technology must be accompanied by implementing new statistical tools that utilize high‐dimensional machine learning approaches to process the large amount of data obtainable from multiparametric assays. Machine learning approaches have already been applied to liquid biopsy biomarkers to identify ovarian cancer patients using neural networks [170]. Nevertheless, the most advanced methods applied to CTC assays were mainly dedicated to the identification and enumeration of cells [171]. Machine learning approaches offer an opportunity to discover cancer‐specific signatures in CTCs, exploiting publicly available data sets from research networks (e.g., TCGA) for simulation experiments. In addition, adopting machine learning strategies can reduce the cost of CTC assays by reducing the depth of analysis required (e.g., depth of sequencing), as well as the quantity of reagents needed. Admittedly, the recent popularity of machine learning programmes has raised ethical and legal concerns. Addressing these issues necessitates either the use of privacy‐oriented algorithms that do not disclose private clinical data or the future availability of machine learning programmes in research institutes to avoid sharing the data with third parties.

The next frontier for CTC‐based tests for resistance monitoring is to address the need for predictive biomarkers for immunotherapy. Immune checkpoint inhibitors have offered the most durable response in NSCLC and melanoma, although it is still difficult to predict the tumor response to most of these inhibitors. This outlook is actually based on a recent report which demonstrated that expansion of T‐cell clones within the tumor is paralleled by their expansion in peripheral blood, which can assist in predicting tumor infiltration and therapeutic response [172]. Consistent with this finding, two recent reports have demonstrated the presence of large numbers of T‐cell clones in the peripheral blood of melanoma patients responding to immune checkpoint blockade [173, 174].

Whatever the clinical question that needs to be addressed or the CTC profiling technology, CTC‐based assays would need to go through thousands of tests for validation to ensure that the assay can respond to the clinical question with sufficient statistical significance. If the CTC assay is based on a microfluidic device, then these devices must be amenable to large scale production at reasonable cost to generate assays that are highly reproducible. A sample‐to‐answer microfluidic system would be highly amenable to clinical practice since it can improve the processing throughput and eliminate the need for manual handling of samples. Several companies are currently developing integrated platforms that combine sorting and downstream analysis.

Another major challenge that still faces the approval of liquid biopsy tests is that tissue biopsies are still adopted by the regulatory agencies as the gold standard. However, the value of tissue biopsies as a reference is questionable because CTCs can be derived from metastatic lesions that were not biopsied. In addition, occult metastases, which are not detectable by current imaging techniques, might contribute to the pool of CTCs in patients. Thus, CTCs might reveal different molecular landscapes than the reference primary tumor, leading to test failure.

To conclude, future CTC assay developments will require orchestrated efforts from preclinical and clinical researchers, spanning a range of disciplines such as basic biology, molecular biology, assay technology, and data analytics. Although the CTC tools we have in hand should keep us busy for the near future, it is up to the next generation of CTC assays to devise solutions that can revolutionize the practice of precision oncology and clinical management of cancer patients.

## Conflict of interest

The authors declare no conflict of interest.
